# A Case of an Insufficiency Fracture of the Medial Proximal Tibia Secondary to Osteomalacia Associated with Long-Term Saccharated Ferric Oxide Administration

**DOI:** 10.1155/2017/1675654

**Published:** 2017-07-04

**Authors:** Daichi Ishimaru, Hiroshi Sumi

**Affiliations:** ^1^Department of Orthopaedic Surgery, Gujo Municipal Hospital, 1261 Shimatani, Hachiman, Gujo, Gifu, Japan; ^2^Department of Orthopaedic Surgery, Sumi Memorial Hospital, 2-1 Shirotori, Gujo, Gifu, Japan

## Abstract

This article presents a case of insufficiency fracture of medial proximal tibia caused by long-term administration of saccharated ferric oxide (SFO) in a 77-year-old female. In this case, 2-year administration of SFO for iron deficit anemia induced hypophosphatemic osteomalacia and finally resulted in an insufficiency fracture of medial proximal tibia. Hypophosphatemia and pain due to the insufficiency fracture were recovered promptly by withdrawing SFO administration and rest. This case represented varus deformity of the knee associated with osteoarthritis, which may also cause the insufficiency fracture of medial proximal tibia in addition to osteomalacia due to long-term administration of SFO. Long-term SFO administration should be avoided because of a definitive risk of osteomalacia and fragile fracture.

## 1. Introduction

Insufficiency fracture is an incomplete fracture caused by repeated tensile or compressive stresses, and generally osteoporosis is one of the most common causes for insufficiency fracture. Herein, we experienced a rare case of insufficiency fracture due to hypophosphatemic osteomalacia, which was caused by long-term administration of saccharated ferric oxide (SFO). SFO is a colloidal preparation of iron with maltose [Fe(OH)3*m*(C12H22O11)*n*] and is widely administered to patients with iron deficient anemia in Japan. To date, long-term administration of SFO induced hypophosphatemia has been reported to be a relatively rare cause of osteomalacia in Japan [1–3]. Herein, we report the case that long-term SFO administration induced hypophosphatemic osteomalacia with the insufficient fracture.

## 2. Case Report

**Figure 1:**
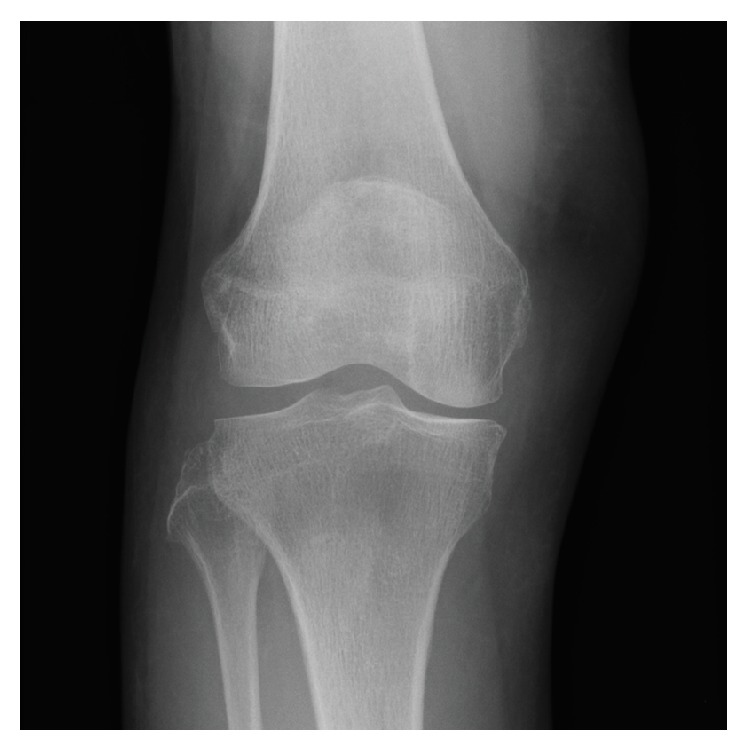
The anteroposterior radiograph of right knee at the onset showing no abnormal finding.

**Figure 2:**
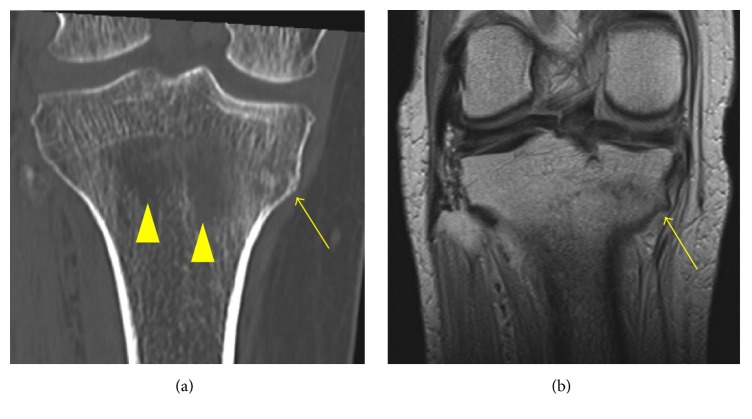
(a) Coronal CT imaging of right knee shows irregularity of cortex in the proximal medial tibia (yellow arrow) with trabecular bone resorption in the central part of the proximal tibia (yellow arrow heads), which indicates osteomalacia. (b) Coronal T1 weighted MR imaging of right knee shows linear low intensity lesion at the proximal medial tibia (yellow arrow), which indicates insufficiency fracture.

**Figure 3:**
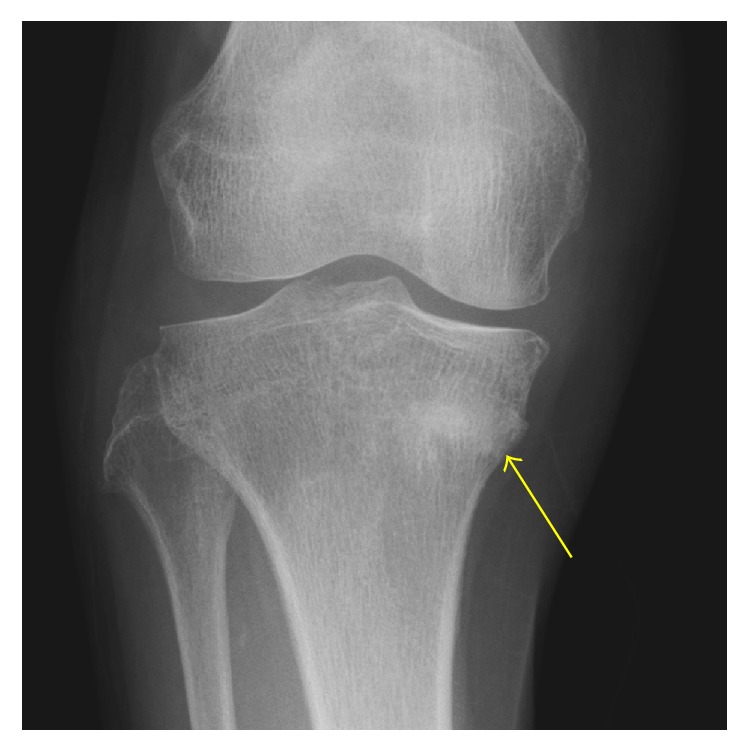
The anteroposterior radiograph of right knee 4 months after the onset showing the bone union of the insufficiency fracture (yellow arrow).

77-year-old female, who complained of right knee pain without an episode of trauma, consulted our hospital. She had past history of iron deficient anemia due to duodenum ulcer, which has been treated by 80 mg/week of intravenous SFO administration for approximately 2 years as she could not take oral iron supplements because of the gastrointestinal side effect, whereas she had no history of steroid administration, diabetes, malignant disease, and customary drinking. In consulting our hospital, the right knee exhibited a slight varus deformity, and the part of medial proximal tibia was swollen. Radiograph of the right knee showed no obvious findings of fracture but the appearance of bone atrophy in the proximal tibia (Figure 1). In computed tomography (CT) imaging of right knee, trabecular bone structure in the central part of the proximal tibia apparently was resorbed, which indicated osteomalacia, and medial cortex of the tibia was partly irregular (Figure 2(a)). Magnetic resonance (MR) imaging could reveal the fracture line of right medial proximal tibia (Figure 2(b)). According to the result of blood examination, it showed slight anemia (haemoglobin 9.5 g/dl, mean corpuscular volume 103.0 fL) and hypophosphatemia (serum phosphorus 1.7 mg/dl), whereas serum calcium and intact parathyroid hormone were in the normal range (8.8 mg/dl and 35 pg/ml, resp.). The level of alkaline phosphatase ranged high (507 IU/l). Dual-energy X-ray absorptiometry of the lumbar vertebrae and femoral neck showed that bone density of each part was preserved in a *T*-score of 107% and 73%, respectively (bone mineral density: lumbar vertebrae 1.081 g/cm^2^ and femoral neck 0.576 g/cm^2^). Overall, an insufficient fracture of medial proximal tibia due to osteomalacia associated with long-term SFO was suspected based on the following facts: no traumatic episode, the history of prolonged SFO administration, blood data including of hypophosphatemia, and radiographic imaging studies. After admission, she was treated by withdrawing SFO administration and using subcutaneous injection of teriparatide [human parathyroid hormone (1–34)] under no weight bearing of right lower extremity. Four weeks after the onset, serum phosphorus was improved in the normal range (3.9 mg/dl), and right knee pain gradually got reduced. 12 weeks after the onset, the radiograph of right knee showed bone formation around the fracture line, and she was allowed to walk with full-weight bearing of right extremity. Six months after the onset, she could walk without right knee pain, and radiographically the fragile fracture was united (Figure 3).

## 3. Discussion

In this time, we experienced the case of insufficiency fracture in medial proximal tibia of the knee, which was caused by 2-year intravenous SFO administration induced hypophosphatemic osteomalacia.

Short period administration of intravenous SFO has been reported to be useful treatment for postoperative anemia and also reduces a risk of postoperative infection or total amount of blood transfusion [4, 5], whereas aimlessly long-term administration of intravenous SFO has a definitively risk of complication including hypophosphatemic osteomalacia [3, 6, 7]. In our case, the trabecular bone absorption and the fracture in the tibia were confirmed by CT and MRI. prolonged intravenous SFO administration induced hypophosphatemic osteomalacia with insufficiency fracture of the proximal tibia. Recently, prolonged intravenous SFO induced hypophosphatemic osteomalacia has been reported to be associated with fibroblast growth factor 23 (FGF-23), which has the critical factor of maintaining serum phosphate homeostasis via suppression of 25-hydroxyvitamin D activation and promotion of phosphaturia, and the patient exhibited high serum FGF 23 levels [8]. In our case, although serum FGF-23 was not estimated during the admission, the diagnosis of insufficiency fracture due to intravenous SFO induced osteomalacia was comprehensively made based on the history of prolonged intravenous SFO administration, occurrence of the fracture without traumatic episode, the result of hypophosphatemia in the blood evaluation, and the finding of CT images showing remarkable trabecular bone resorption around the fracture site. In addition, the fact that hypophosphatemia got improved promptly after discontinuing intravenous SFO administration supports the diagnosis of the insufficient fracture due to hypophosphatemic osteomalacia because withdrawing SFO administration has been reported to improve promptly hypophosphatemia and osteomalacia due to prolonged intravenous SFO [1].

This fracture also might be associated with mechanical loading on the knee. In the onset of the fracture, her knee joint had showed varus deformity accompanied with knee osteoarthritis, radiographically with 186 degrees of femorotibial angle at standing position. In addition to osteomalacia due to prolonged intravenous SFO, repeated loading stress on the knee joint while walking focused on the medial side of tibial plate by varus deformity of the knee joint, which might result in insufficiency fracture in the proximal medial tibia. In addition, this situation that the patient had the fracture of medial cortex in the tibia with varus knee had a dynamic disadvantage for bone union. Therefore, we used teriparatide to progress bone union of the fracture instead of phosphate salt and active vitamin D.

We experienced the rare case of the insufficient fracture of medial proximal tibia due to prolonged intravenous SFO administration. Suspension of intravenous SFO could promptly improve the hypophosphatemia, and the insufficiency fracture got united with limiting weight bearing toward the right extremity and administration of teriparatide. Long-term SFO administration should be avoided because of a definitive risk of osteomalacia and fragile fracture.

## References

[1] Sato K., Shiraki M. (1998). Saccharated ferric oxide-induced osteomalacia in Japan: iron-induced osteopathy due to nephropathy. *Endocrine Journal*.

[2] Okada M., Imamura K., Iida M., Fuchigami T., Omae T. (1983). Hypophosphatemia induced by intravenous administration of saccharated iron oxide. *Klinische Wochenschrift*.

[3] Sato K., Nohtomi K., Demura H. (1997). Saccharated ferric oxide (SFO)-Induced osteomalacia: in vitro inhibition by SFO of bone formation and 125-dihydroxy-vitamin D production in renal tubules. *Bone*.

[4] Cuenca J., García-Erce J. A., Martínez A. A., Solano V. M., Molina J., Muñoz M. (2005). Role of parenteral iron in the management of anaemia in the elderly patient undergoing displaced subcapital hip fracture repair: Preliminary data. *Archives of Orthopaedic and Trauma Surgery*.

[5] Serrano-Trenas J. A., Ugalde P. F., Cabello L. M., Chofles L. C., Lázaro P. S., Benítez P. C. (2011). Role of perioperative intravenous iron therapy in elderly hip fracture patients: A single-center randomized controlled trial. *Transfusion*.

[6] Shimizu Y., Tada Y., Yamauchi M. (2009). Hypophosphatemia induced by intravenous administration of saccharated ferric oxide. Another form of FGF23-related hypophosphatemia. *Bone*.

[7] Suzuki A., Ohoike H., Matsuoka Y., Irimajiri S. (1993). Iatrogenic osteomalacia caused by intravenous administration of saccharated ferric oxide. *American Journal of Hematology*.

[8] Yamamoto S., Okada Y., Mori H., Fukumoto S., Tanaka Y. (2012). Fibroblast growth factor 23-related osteomalacia caused by the prolonged administration of saccharated ferric oxide. *Internal Medicine*.

